# Prevalence of Enteric Pathogens and Antibiotic Resistance: Results of a Six-Year Active Surveillance Study on Patients Admitted to a Teaching Hospital

**DOI:** 10.3390/antibiotics13080726

**Published:** 2024-08-02

**Authors:** Nadia Marascio, Grazia Pavia, Brunella Brescia, Concetta Riillo, Giorgio Settimo Barreca, Luigia Gallo, Cinzia Peronace, Simona Gigliotti, Marta Pantanella, Angelo Giuseppe Lamberti, Giovanni Matera, Angela Quirino

**Affiliations:** Unit of Clinical Microbiology, Department of Health Sciences, “Magna Græcia” University, 88100 Catanzaro, Italy; nmarascio@unicz.it (N.M.); graziapavia@unicz.it (G.P.); brunella.brescia@studenti.unicz.it (B.B.); criillo@unicz.it (C.R.); gbarreca@unicz.it (G.S.B.); l.gallo@materdominiaou.it (L.G.); cinziaperonace@hotmail.it (C.P.); s.gigliotti@unicz.it (S.G.); marta.pantanella@studenti.unicz.it (M.P.); alambert@unicz.it (A.G.L.); quirino@unicz.it (A.Q.)

**Keywords:** gastrointestinal infections, enteric pathogens, epidemiological trend, hospitalized patients, antibiotic susceptibility

## Abstract

Background: Acute Infectious Diarrhea (AID) and the short- and long-term complications associated with it are major causes of hospitalization worldwide. In Italy, due to a lack of robust surveillance programs, only limited data has been collected on their prevalence and circulation. This study aims to evaluate the resistance pattern of enteric pathogens and their epidemiological trends over a six-year period. Methods: This cross-sectional retrospective study was conducted from January 2018 to December 2023. Stool samples were analyzed during routine diagnosis with culture methods, syndromic molecular tests, and enzyme immunoassay. Results: Bacteria were the most isolated enteric pathogens (62.2%), followed by fungi (29.0%), viruses (8.2%), and parasites (0.6%). Most bacteria were isolated from outpatients (29.5%) and from patients in the Oncology ward (26.2%). The most prevalent target was EPEC (11.1%), followed by *C. difficile* toxin A/B-producing strains (8.3%), *C. jejuni* (2.5%), and *S. enterica*, (1%.). Norovirus and *Candida* spp. were the most prevalent in pediatric patients (6.5% and 39.6%, respectively). In the last years, enteric pathogens have been a frequent cause of infections characterized by a problematic resistance to common antimicrobials. In our study, *S. enterica* showed resistance to amikacin, gentamicin, ampicillin, levofloxacin, and ciprofloxacin. *C. jejuni* was susceptible to all tested drugs. Conclusion: Timely notification of gastroenteric infections is crucial in identifying potential outbreak sources and ensuring strict adherence to food safety and hygiene practices, so as to protect the most vulnerable populations. The present study offers insights into the epidemiological characteristics and the antibiotic susceptibility of the main enteric AID pathogens in order to implement infection control measures in health care settings.

## 1. Introduction

The spread of enteric pathogen infections differs between developed and developing countries in relation to health care infrastructure, economic disparities, food safety, and climate [1]. In the last years, the COVID-19 pandemic has also affected the diffusion of common pathogens, including enteric ones, leading to a global adoption of nonpharmaceutical measures such as social distancing and hand cleaning [2]. Gastroenteric infections, characterized by gastrointestinal tract inflammation, are a significant cause of morbidity worldwide, resulting in 1.6 million deaths annually [3]. Acute Infectious Diarrhea (AID) and the short- and long-term complications associated with it are responsible for symptoms ranging from mild discomfort to severe and life-threatening illnesses requiring hospitalization [3,4]. AID causes dehydration and other serious conditions, especially in vulnerable populations such as children, the elderly, and immunocompromised individuals [3].

Despite AID being an issue in Italy, Italian research on this disease is rather scarce. Only a few studies have been published on the different types of pathogens involved in AID; however, when taking foodborne disease into account, the amount of germane research increases. Surveillance of AID is of paramount importance to achieving affordable infection control and strengthening prevention campaigns [5]. However, some studies dealt with both bacterial and viral infections [5], and some focused only on viral ones [6], while others focused on protozoan infections in humans [7,8]. Among human gastrointestinal diseases, campylobacteriosis, listeriosis, and salmonellosis were the ones detected in an Italian study [5]. Moreover, in another investigation, several Salmonella strains were found and processed in order to study their antibiotic resistance [9]. Several different protozoan species were found in both pediatric and adult patients’ stool specimens in two studies carried out in Italy [7,8]. The following viral pathogens were found in pediatric patients with gastroenteritis: adenovirus, aichivirus, astrovirus, enterovirus, human parechovirus, norovirus, rotavirus, sapovirus, and salivirus [10]. A few investigations also addressed the antibiotic resistance of enteric bacterial pathogens [9]. Patients from the Oncological and hematological wards, as well as those from the Intensive Care and Neonatal Units, display a high risk of developing infections as well as bacterial resistance to several widely used antibiotics [11]. A correct antibiotic therapy, including both empiric and tailored drug selections, is necessary in order to enhance the efficiency of antibiotics. As such, in spite of the aforementioned studies, a truly thorough and updated epidemiological study on viral, bacterial, and protozoan AID has not yet been conducted.

A broad spectrum of pathogens is implicated in AID, each of which demonstrates distinct epidemiological patterns, seasonality variations, and different age distribution. *Salmonella enterica*, *Campylobacter jejuni*, *Clostridioides difficile* (toxin A/B-producing strains), *Yersinia enterocolitica*, and various serotypes of *Escherichia coli*, including Enteroaggregative *E. coli* (EAEC), Enterotoxigenic *E. coli* (ETEC), Enteropathogenic *E. coli* (EPEC), and Shiga-like toxin-producing *E. coli* (STEC), are the most identified microorganisms [12,13]. According to the European Centre for Disease Prevention and Control (ECDC), *C. jejuni* and *S. enterica* serovars *S. typhimurium* and *S. enteritidis* are the leading causes of bacterial gastroenteritis in Europe [12,13]. Salmonellosis, with 65,208 confirmed cases, affects both pediatric and adult populations, with severe systemic illness occurring particularly in immunocompromised individuals [13]. In addition, the *C. difficile* toxin A/B-producing strain is a significant cause of gastroenteritis in adults, particularly in hospitalized patients and in those undergoing antibiotic therapy [14]. Yersiniosis, with a total of 7919 confirmed cases, most of which have been reported in children under the age of five, is the third most reported zoonosis in humans, followed by STEC infection [12]. Among the several serotypes of *E. coli*, STEC is associated with a more severe disease course and with increased complications compared to other serotypes, such as hemolytic uremic syndrome (HUS) [15]. In 2022, these pathogens accounted for a significant proportion of reported foodborne illnesses, highlighting the critical need for rigorous food safety measures and hygiene practices in order to mitigate their impact [13]. Several viral pathogens, including Norovirus, Sapovirus, Rotavirus, Astrovirus, and Adenovirus, are also well-known agents of AID [3,16,17]. Prior to the introduction of a vaccine for it, Rotavirus was responsible for substantial morbidity and mortality among European children. After the introduction of the vaccine, a significant decline in Rotavirus-associated hospitalizations and a decrease in severe cases have been documented [18]. Norovirus is the most common cause of gastroenteritis among all age groups. Despite its self-limiting nature, this virus can cause severe dehydration, particularly in vulnerable populations such as the elderly and the immunocompromised [19,20]. Adenoviruses, particularly types 40 and 41, and Astrovirus are notable causes of pediatric gastroenteritis [21], though less common if compared to Rotavirus and Norovirus. Both viruses are less frequently associated with severe disease [22,23]. Additionally, enteric infections caused by fungi and parasites, as opportunistic pathogens, can play a critical role in the epidemiology of gastroenteric diseases, mainly associated with prolonged use of antibiotics, immunodeficiency, diabetes, and dysbiosis [24,25]. 

Enteric pathogen control is a challenging effort due to these pathogens persisting in the environment for a long time. Since most pathogens cause similar symptoms, delayed or missed identification can have an impact on patient management and on general population spread. Standard microbiological culture and enzyme immunoassays in association with molecular assays have improved the sensitivity and the detection of multiple enteric pathogens, reducing the turnaround time [26]. The aim of our study was to evaluate the epidemiology and the antibiotic susceptibility of the main enteric pathogens over the course of six years among patients with suspected AID admitted to a teaching hospital in southern Italy. We evaluated pathogens’ prevalence, including their trends across different seasons and age groups, in order to conduct comprehensive surveillance and to provide more information for effective public health interventions.

## 2. Results

### 2.1. Epidemiological Survey

The demographic characteristics of 2863 patients screened for AID and seasonality are reported in Table 1. Overall, 97.8% were Italian, with a median age of 49 years (IQR 44). Most screened patients were registered in 2023 (947, 33.1%), mostly in the autumn season (766/694, 26.8%) (Table 1). Over the course of the considered time span, the prevalence rate of positive samples was found to be 23% in 2018 and 2020, while in 2022 and 2023, the rate showed a percentage of 25% and 24%, respectively. Finally, a substantial decrease in positive cases (9.2%) was observed in 2021.

With 694 positive samples, bacteria were the most common isolated enteric etiological agents (432/694, 62.2%), followed by fungi (201/694, 29.0%), viruses (57/694, 8.2%), and parasites (4/694, 0.6%) (Table 2). Most bacteria were isolated from outpatients (205/694, 29.5%) and from patients in the Oncology (182/694, 26.2%) and Gastroenterology (92/694, 13.3%) Units (Table 2).

**Figure 1:**
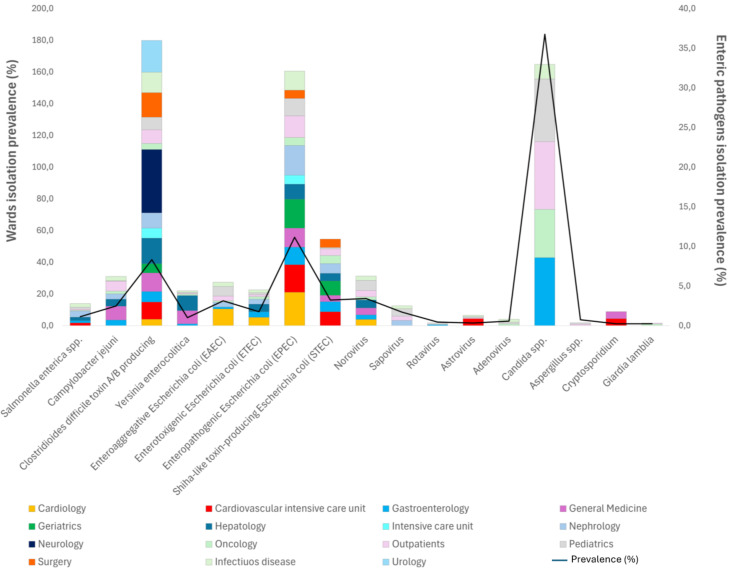
Prevalence of enteric pathogens among different hospital units. The number of enteric pathogens isolated in the pediatric ward refers only to the 2022–2023 biennium.

**Figure 2:**
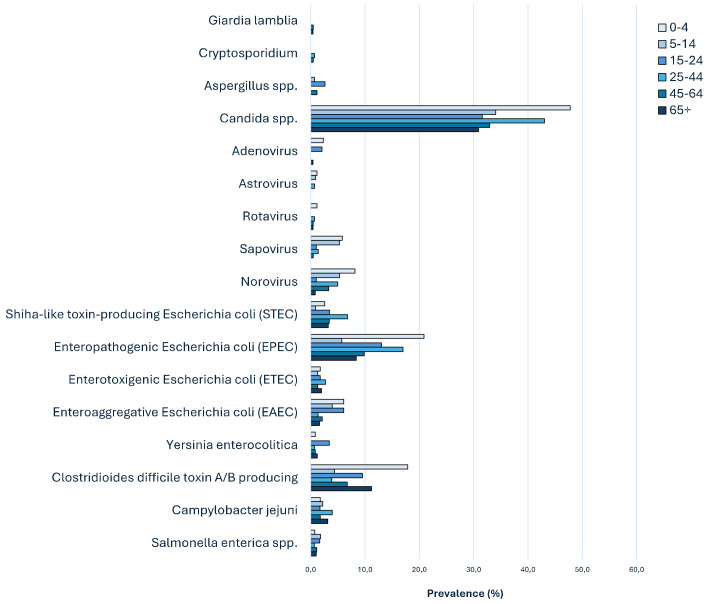
Distribution and circulation of enteric pathogens among different age groups.

The prevalence rate was obtained by calculating the ratio between the number of positive isolates and the total number of requests for each enteric pathogen, according to the different wards. The most prevalent bacteria was EPEC (121/1086, 11.1%), mostly found in the Cardiology (4/19, 21%), Nephrology (6/32, 18.8%), and Cardiovascular Intensive Care (4/23, 17.4%) Units, followed by *C. difficile* toxin A/B-producing strains (158/1896, 8.3%), STEC (35/1086, 3.2%), EAEC (34/1086, 3.13%), *C. jejuni* (27/1091, 2.5%), *S. enterica*, *Y. enterocolitica*, and ETEC with about 1% of prevalence (Figure 1). The circulation of *C. difficile* toxin A/B-producing strains was most prevalent in the Neurology (2/5, 40%), Hepatology (5/31, 16.1%), Surgery (9/58, 16.5%), Infectious Disease (29/225, 12.9%), and Cardiovascular Intensive Care (5/46, 11%) wards (Figure 1). However, Norovirus was the most prevalent (30/875, 3.4%) and was mostly found in Pediatrics (11/170, 6.5%), followed by Sapovirus (15/875, 1.7%), while Astrovirus, Rotavirus, and Adenovirus had a prevalence of about 0.6% (Figure 2). With regard to opportunistic infections, *Candida* spp. was the most prevalent (197/536, 36.7%) and was most commonly found in pediatric patients, patients from the Gastroenterology Unit, and outpatients (Figure 1). *Aspergillus* spp. infection had a prevalence of 0.8%, mainly in pediatric patients and outpatients (0.9%). Only 0.2% of AID cases was observed to be caused by parasitic infection in the Oncology and Cardiovascular Intensive Care Units.

**Figure 3:**
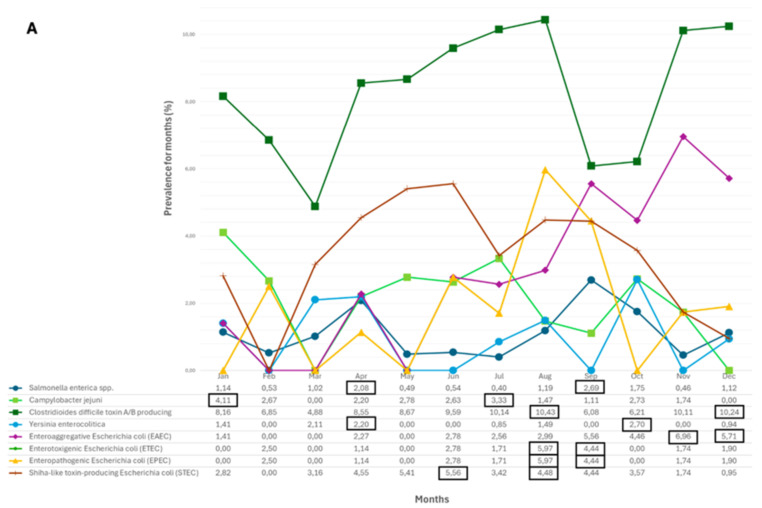

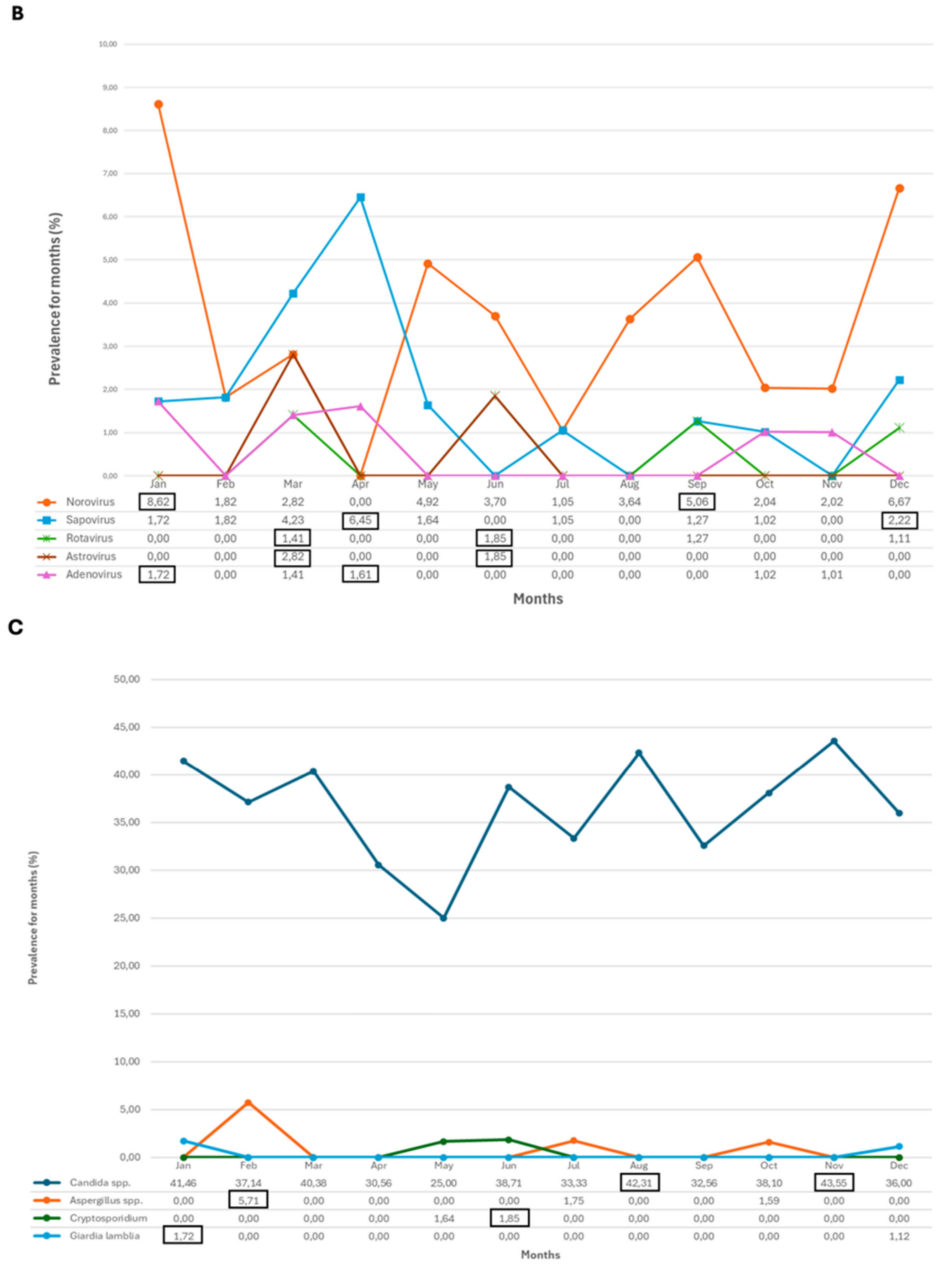
Seasonality trends of different enteric pathogens. Bacterial (**A**), Viral (**B**), and Fungal and parasitic (**C**) pathogens. The black boxes at the bottom highlight the peaks of highest prevalence (%) for each microorganism in relation to the month.

EPEC showed a higher prevalence in early childhood, particularly in the 0–4 age group (24/115, 20.8%), followed by the 25–44 (25/147, 17%), the 15–24 (15/115, 13%), the 45–64 (23/233, 9.9%), the over-65 (21/249, 8.4%), and the 5–14 (13/227, 5.7%) age groups. *C. difficile* toxin A/B-producing strains were most prevalent in the 0–4 age group (22/123, 17.8%), followed by the over-65 (65/580, 11.21%), the 15–24 (17/178, 9.6%), the 45–64 (32/477, 7.7%), the 5–14 (10/228, 4.3%), and the 25–44 (12/310, 3.9%) age groups. EAEC showed a prevalence of 6.1% in both the 0–4 and 15–24 age groups, followed by the 5–14 (9/227, 4%) and the 45–64 (5/233, 2.2%) age groups, while the 25–44 and the over-65 age groups had a prevalence of about 1% (Figure 2). STEC infection was most prevalent among adults, including the 25–44 age group (10/147, 6.8%), followed by all other age groups with around 3% frequency (Figure 2). *C. jejuni* circulated mainly in the 25–44 (6/151, 4%) and the over-65 (8/253, 3.2%) age groups, followed by the others with a prevalence of around 2% (Figure 3). *S. enterica* infection was observed in the 0–4 and 5–14 age groups with a percentage of 1.6–1.8%, followed by the others with frequencies ranging from 0.7 to 1.1% (Figure 2). *Y. enterocolitica* circulation was observed the most in the 15–24 age group (4/117, 3.4%), followed by the oldest group (3/247, 1.2%) (Figure 2). ETEC showed the highest prevalence (2 to 2.7%) in adults from the 25–44 and the over-65 age groups, followed by the others ranging from 1.7 to 1.3% (Figure 2). Norovirus was most prevalent in the youngest groups, particularly in the 0–4 (7/86, 8.1%) age group, followed by the 5–14 (6/113) and the 25–44 (7/140) age groups with a frequency of 5%. A similar age distribution was observed for Astrovirus, Rotavirus, and Adenovirus. *Candida* spp. showed a high prevalence in all age groups, but it was particularly common in the 0–4 age group (43/90, 47.8%), while *Aspergillus* spp. infection was most commonly observed in patients belonging to the 15–24 age group (2/76, 2.6%). *Cryptosporidium* was isolated from two 39- and 54-year-old patients, while *G. lamblia* was isolated from two 52- and 75-year-old patients (Figure 2).

With regard to seasonal trends, Figure 3 shows the prevalence of each enteric pathogen, subdivided into bacterial (Figure 3A), viral (Figure 3B), and fungal/parasitic pathogens (Figure 3C). Among the bacteria, *S. enterica* and *Y. enterocolitica* exhibit two seasonal peaks, primarily in spring (April) and in autumn (September-October). *C. jejuni* and *C. difficile* toxin A/B-producing strains have a higher prevalence in winter (December–January) and summer (July–August). ETEC and EPEC primarily circulate in summer (August) and autumn (September). The other two *E. coli* serotypes, EAEC and STEC, show a respective higher prevalence in winter (November–December) and summer (June–August) (Figure 3A). As for the viruses (Figure 3B), Norovirus circulates exclusively in winter (December–January), while Sapovirus and Adenovirus are present both in winter (December–January) and in spring (April). Rotavirus and Adenovirus show a spike in frequency that takes place in March (1.41%) and June (1.85%), respectively. The spikes in frequency (1.85%) of Astrovirus and Rotavirus overlap in June. *Candida* spp., which exhibits a significantly higher prevalence compared to the other enteric opportunistic pathogens evaluated, shows two peaks that take place in summer (August) and autumn (November) (Figure 3C).

### 2.2. Antimicrobial Resistance Pattern

The antimicrobial resistance pattern was evaluated for *S. enterica* spp., *C. jejuni*, and *Y. enterocolitica*. *S. enterica* isolates were all meropenem sensitive and showed the following resistances: amikacin (6/27), gentamicin (5/27), ampicillin (3/27), levofloxacin and ciprofloxacin (2/27), and cefotaxime (1/27). *C. jejuni* was susceptible to ciprofloxacin, erythromycin, and tetracycline, while *Y. enterocolitica* was susceptible to all tested drugs according to the bioMérieux protocol: amikacin, amoxicillin and clavulanic acid, ampicillin, cefepime, cefotaxime, ciprofloxacin, levofloxacin, ertapenem, fosfomycin, gentamicin, meropenem, and piperacillin/tazobactam.

## 3. Discussion

The prevalence of enteric pathogens is modulated by age, social, and geographic factors, and, in the last years, the dynamic trend of the SARS-CoV-2 spread has also played a role [2,27]. Several countries, including Italy, lack comprehensive data on infection seasonality, enteric infectious agents, and their epidemiological characteristics in different age populations [28]. This gap is largely attributable to the lack of robust surveillance programs, which hampers effective monitoring and control measures. Antimicrobial treatment is not routinely recommended, as most human cases resolve on their own within a few days, but it may be recommended for symptomatic patients, particularly immunocompromised individuals. Moreover, published data on AID epidemiology conducted in hospital settings focused on selected enteric pathogens, thereby providing an incomplete overview of this population [16,18,21,23]. The present study offers extensive insight into the epidemiological characteristics and the antibiotic susceptibility of primarily enteric pathogens linked to AID in patients admitted to a teaching hospital in southern Italy. Routinely collected stool samples were analyzed by conventional and syndromic molecular assays to assess the prevalence of the pathogens responsible for gastroenteritis. Recent advances in diagnostic technology, such as multiplex molecular-based methods, have enhanced the clinical management of patients [1].

Over a six-year period, a total of 2863 patients were tested for suspected AID. Even if the number of requests was similar in both the pre-pandemic and the pandemic eras (about 12%), an increased trend was observed in 2022 (18.8%) and 2023 (33.1%) during the late pandemic period. In particular, the prevalence rate of positive patients was 23% in the pre- and early pandemic (2018–2020), and it ranged from 25% to 24% in the late pandemic era (2022–2023). In 2021, during the strictest Italian lockdown, the prevalence was very low (9.2%); this was probably related to a series of control measures, such as the closure of commercial activities and the implementation of smart working put into place by the Italian government to hinder the transmission of COVID-19, which resulted in a reduction of infectious disease cases [29]. Epidemiological data and recent European reports referred to a different prevalence of enteric pathogens according to geographical region [12,13,30,31]. Although most recent investigations on hospital-acquired infections have been carried out on COVID-19 patients, our data indicate that enteric pathogens related to health care-associated infections slightly increased from the pre- to the post-pandemic period, a result that is in accordance with a recent paper published on hospital-acquired infections [32]. Health care management is influenced by the health status of the patients (i.e., immunosuppression, cancer, and chronic respiratory diseases), increasing the lengths of hospital stays compared with patients without comorbidities [33,34]. Enteric pathogens such as *Salmonella*, *C. jejuni*, Rotavirus, and *C. difficile* are common diseases leading to hospitalization and mortality in children and the elderly [33,34].

Our primary findings reveal that bacteria were the most frequently isolated enteric pathogens, accounting for 62.2% of total cases, followed by fungi (29.0%), viruses (8.2%), and parasites with a prevalence of 0.6%. In line with different studies conducted in hospitalized subjects and outpatients, the most commonly detected pathogens in patients with diarrhea were bacteria, followed by viruses and parasites [30,31]. Moreover, the percentage of infections was significantly higher among outpatients (29.5%), oncologic individuals (26.2%), and patients with gastroenteric chronic diseases (13.3%). The elevated number of isolated fungal opportunistic pathogens is probably due to the high prevalence of enteric infections among frail patients and subjects with gastroenteric chronic diseases. In such cases, the gut dysbiosis caused by an imbalance or a disruption in the composition and function of the gut microbiota could potentially impact susceptibility to AID [24,25]. As previously reported, factors like chemotherapy, radiation, and chronic inflammation, such as Inflammatory Bowel Disease (IBD), lead to a reduction in microbial diversity, the loss of beneficial microbes, and the triggering of impaired host immune responses [24,35,36,37]. 

Among bacteria, EPEC was the most prevalent pathogen (11.1%), followed by *C. difficile* toxin A/B-producing strains (8.3%). Notably, their circulation was detected among patients with chronic diseases, such as neurological disorders, liver dysfunction, other infections such as persistent respiratory illnesses, and severe clinical conditions. Additionally, we found the following prevalence: STEC (3.2%), EAEC (3.13%), *C. jejuni* (2.5%), and *S. enterica*, *Y. enterocolitica*, and ETEC, each at about 1%. This epidemiological scenario may be linked to the characteristics of the studied population. In hospitalized patients, several factors, such as compromised immune systems, prolonged or intensive antibiotic use, and underlying health conditions, could make them more susceptible to EPEC and *C. difficile* toxin A/B-producing strain infection [25,38,39,40]. Previous studies revealed the association between EPEC enteric infections and an increase in inflammation markers, such as the level of fecal calprotectin [41,42]. The presence of an alteration in microbiota composition in association with pro-inflammatory activated pathways might contribute to the pathophysiology of EPEC. Moreover, prolonged hospital stays increase the likelihood of being exposed to these pathogens and their spread [43]. Indeed, the incidence of CDI is notably high in health care settings across many European countries [44,45], posing a considerable burden on health care systems and underscoring the importance of infection control practices and antimicrobial stewardship. With regard to age- and seasonality-related prevalence patterns, our findings are in line with data from the literature and ECDC/EFSA reports [12,13]. EPEC was most common in early childhood, circulating primarily in summer and secondarily in autumn. The heightened susceptibility in this age group may result from multiple factors, including host susceptibility related to the child’s age, breastfeeding, and nutritional and immunological status [25,46,47]. The severity of infection and the risk of complications are higher in infants, who are, therefore, in need of preventive measures and effective therapies. Moreover, warm temperatures enhance the survival and proliferation of *E. coli* in the environment. Increased food consumption patterns during the warmer seasons, such as eating raw or undercooked foods, can heighten the risk of EPEC transmission [12,13,48]. *C. difficile* toxin A/B-producing strains showed the highest prevalence in the 0–4 and in the over-65 age groups, with greater circulation in winter and summer. The risk of CDI increases significantly with age, as elderly individuals are more susceptible due to their weakened immune systems, higher rates of comorbidities, and increased exposure to health care settings [44,45,49]. However, data on the association between seasonality and CDI risk are unclear and contradictory, possibly reflecting geographical variations in antibiotic use patterns, infection control practices, and hospitalization rates. In accordance with European data [12,48], we observed an age-related trend for EAEC and *S. enterica* infections, as they were mainly detected in pediatric patients, with peaks in winter/summer and spring/autumn, respectively. In our study, we found two non-typhoidal Salmonella strains resistant to both levofloxacin and ciprofloxacin drugs. Fluoroquinolones, as well as cephalosporins, are the first-line antimicrobials to treat salmonellosis infection; therefore, increased resistance to them significantly restricted treatment options, which impacted clinical outcomes [50]. Conversely, STEC, ETEC, and *C. jejuni* showed a higher prevalence among adults, with peaks in winter and summer. In Italy, *Campylobacter* resistance to fluoroquinolones and tetracyclines has been found to be decreasing; in line with this data, we found only susceptible isolates [51]. *Y. enterocolitica* was most common in young adults aged 15–24 years and in the oldest age group, primarily in spring and autumn. Conversely, *Candida* spp. exhibited high prevalence across all age groups, particularly in the 0–4 and 25–44 age groups, mainly in the warm and/or autumn months. *Candida* species are a common component of the resident microbiota of the human gastrointestinal (GI) tract [52]. It has been reported that GI *Candida* spp. colonization can play a beneficial role in the mammalian host’s health by inhibiting the adhesion of pathogens to the mucus layer through interactions with the immune system [53,54]. However, in vivo studies have linked high levels of *Candida* colonization with various gastrointestinal diseases, such as IBD. Its colonization exacerbates inflammatory lesions, while the inflammation itself appears to promote further colonization [52,55]. Moreover, *Candida* can disseminate from the intestine and lead to bloodstream infections, which often result in severe outcomes [56]. Given the high prevalence of enteric *Candida* infections in our hospitalized patient cohort—particularly those in the pediatric and in the oncological wards, as well as those with chronic gastrointestinal conditions—these insights may have significant clinical implications.

The most prevalent enteric viruses were Norovirus (52.6%) and Sapovirus (26.3%), while Astrovirus, Rotavirus, and Adenovirus were detected in 9.0% of cases. Although a similar age distribution was reported for Astrovirus, Rotavirus, and Adenovirus, Norovirus was mainly isolated in pediatric patients aged 0 to 4 years (8.1%). In 2016, Grytdal and colleagues reported the highest prevalence of Norovirus among patients aged 46–65 years, between November and March [57], while we found the highest prevalence between December and January. In different studies, Astrovirus and Sapovirus were estimated to be the major causes of viral gastroenteritis among oncological patients >18 years old (18%), as well as children (<5 years old) affected by acute gastroenteritis with a respective frequency of 5% and 3.4% [58]. The peak prevalence for both viruses was detected in spring (March or April), a finding that concurs with previously published data that takes geographical location into consideration [58,59]. About 2% of children aged 0–4 years were Rotavirus positive, and so were 0.7% of adults aged 25–44 years; as for seasonality-related prevalence, it reached its peak in March and September. Similar data were previously reported in children; however, the highest prevalence was found in adults over 65 years during March and April [57]. Very recently, Rotavirus was found to be more prevalent (25%) in children < 5 years with a 36% hospitalization rate and a peak in autumn and winter [1]. Finally, *Cryptosporidium* and *G. lamblia* infections were detected in a few cases (4/694) and respectively occurred in winter and summer. *Cryptosporidium* spp. and *G. lamblia* were included in the top ten of protozoa with significant public health importance in Europe, including Italy and other countries located in the same geographic area [60]. A recent Italian study found both parasites in ready-to-eat salads and berries. *G. lamblia* was observed in spring, whilst *Cryptosporidium* displayed no seasonal variations [60]. Several pathogens were recognized as the etiology of clinically indistinguishable AID. Enteric pathogens are the most common illnesses in humans and the leading cause of death in special populations, such as immunocompromised and pediatric patients [12,13]. Their spread is a significant public health challenge due to its transmissibility during different seasons, which is related to its dissemination in the environment through contaminated food or water, person-to-person contact, and fecal–oral routes [61]. As such, proactive surveillance of enteric pathogens needs to be performed and notified in the human population and in wastewater to monitor their annual fluctuations and to identify the outbreak or the source of the contamination in a timely fashion.

This observational study included symptomatic patients admitted to the hospital over a six-year period, which limited bias from any gastrointestinal pathogen outbreaks or epidemics. This microbiological study does not include clinical information due to its retrospective and observational design. The missing information on previous antibiotic treatment could give false-negative results due to samples not being properly collected. Additionally, a total of 875/2863 (30.6%) patients were screened using molecular techniques, which may create a bias, especially in the underestimation of virological results.

## 4. Materials and Methods

### 4.1. Study Design

**Figure 4:**
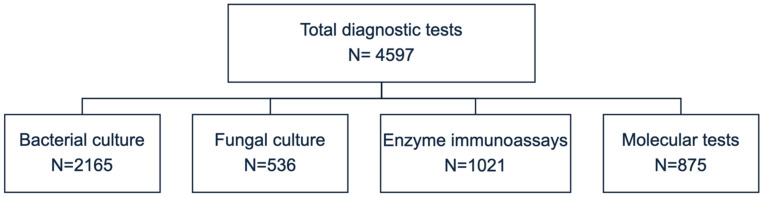
Stool samples tested from 2863 consecutive patients with suspected AID.

Between 1 January, 2018 and 31 December, 2023, a total of 2863 patients (from 0 to 99 years old) admitted to the “R. Dulbecco” University Hospital of Catanzaro were screened for suspected AID. The monocentric study did not include clinical information (e.g., vaccination status, symptoms, and signs), coinfections were not considered, and each pathogen was counted as a single infection. Patients’ demographic information was recorded in an anonymized database at the moment of admittance. The study was conducted in accordance with the Declaration of Helsinki and approved by the Ethics Committee of the Calabria Region (protocol code #130 and date of approval 23 April 2024). Diagnostic tests were performed during hospitalization or during routine diagnosis for outpatients according to clinical query (Figure 4).

### 4.2. Culture and Molecular Diagnostic Methods

The stool specimens were collected over the course of the six years and analyzed fresh. The stool samples were processed according to diagnostic algorithms proposed by the Italian Association of Clinical Microbiologists (AMCLI) [62]. As for the culture method, the fecal sample was inoculated into agar culture media selective for each microorganism and enrichment broth (1:5 dilution). Subsequently, a few drops of the fecal suspension were inoculated into selective agar culture media. For molecular tests on the FilmArray^®^ BioFire^®^ platform (Biomérieux, Florence, Italy), stool samples were re-suspended in Para-Pak^®^ C&S transport solution (Meridian Bioscience Europe, Milan, Italy). for enteric pathogens, as recommended by the manufacturer. Subsequently, 200 μL of fecal suspension was added to the sample buffer within the sample injection vial provided by the FilmArray^®^ BioFire^®^ Gastrointestinal Panel (FA-GP) kit (Biomérieux, Florence, Italy), as recommended by the producer. The Limit of Detection (LoD) for FilmArray^®^ BioFire^®^ Gastrointestinal Panel (FA-GP) analytes (Biomérieux, Florence, Italy) were estimated via dilutions of single-pathogen and multi-pathogen samples (up to four organisms per sample). Detection was equivalent between single-pathogen and multi-pathogen samples, and LoD confirmation testing was performed by spiking one or more organisms into stool samples at the estimated LoD concentration and testing 20 replicates per sample. The LoD concentrations were confirmed on the BIOFIRE 2.0 and BIOFIRE Torch systems with analyte detection in at least 19/20 replicates (≥95%). The LoD concentration for each enteric pathogen species was reported in a BioFire^®^ System Document revised on 7 August 2023.

Stool specimens were tested for the presence of *S. enterica*, *C. jejuni*, *C. difficile* toxin A/B-producing strains, and *Y. enterocolitica* using both culture methods and/or syndromic molecular assay FilmArray^®^ BioFire^®^ Gastrointestinal Panel (FA-GP) (Biomérieux, Florence, Italy), while *E. coli* serotypes were carried out only through FA-GP. Results were available within 1 h. For the diagnosis of *C. difficile* toxin A/B-producing strains, the rapid membrane enzyme immunoassay C. DIFF QUIK CHEK COMPLETE^®^ test (TechLab, Blacksburg, VA, USA) was also performed so as to detect both toxins’ A/B production and glutamate dehydrogenase (GDH), according to the manufacturer’s instructions. Fungal infections were investigated by culture methods. Moreover, enteric viruses such as Norovirus GI/GII, Sapovirus, Rotavirus, Astrovirus, Adenovirus, and parasites including *Cryptosporidium*, *G. lamblia*, *Entamoeba histolytica*, and *Cyclospora cayetanensis* were evaluated using an FA-GP panel.

### 4.3. Antibiotic Resistance Assays

Antimicrobial susceptibility tests (ASTs) were performed using a Vitek^®^2 System (bioMérieux, Florence, Italy), Sensititre System (ThermoFisher Scientific, Waltham, MA, USA), and Disc Diffusion method (only *C. jejuni* isolates were tested), according to the European Committee on Antimicrobial Susceptibility Testing (EUCAST) guidelines. To determine the minimum inhibitory concentration (MIC) of isolated bacterial pathogens, the following drugs were tested: amikacin, gentamicin, ampicillin, levofloxacin, ciprofloxacin, cefotaxime, erythromycin, tetracycline, amoxicillin, clavulanic acid, cefepime, ertapenem, fosfomycin, meropenem, and piperacillin/tazobactam.

## 5. Conclusions

The complex epidemiology of enteric pathogens in AID calls for targeted surveillance and tailored clinical approaches, as well as more effective public health interventions, three factors that can manage and mitigate these infections effectively. Data that include the assessment of AID during symptomatic and asymptomatic periods are limited. In order to track changes in the prevalence of gastroenteric pathogens and in resistance patterns over time, we suggest designing a multicenter, prospective, and longitudinal study, which should include demographic data, clinical symptoms, antimicrobial therapy, vaccination history, and, finally, the outcomes of diseases. Timely notification of gastroenteric infections is crucial in identifying potential outbreak sources and in ensuring strict adherence to food safety and hygiene practices. Knowledge about seasonality- and age-related trends, as well as susceptibility to antibiotics, plays a pivotal role in the development of targeted preventions, such as the implementation of infection control measures in health care settings to protect the most vulnerable populations.

## Figures and Tables

**Table 1:** Patients’ demographic characteristics and their distribution over the course of the six-year study.

Male, n (%)	1415 (49.4)
Female, n (%)	1448 (50.6)
Age in years, median (IQR)	49 (43)
Italian nationality, n (%)	2801 (97.8)
Patients per year, n (%)	
2018	306 (10.7)
2019	356 (12.4)
2020	348 (12.2)
2021	369 (12.9)
2022	537 (18.8)
2023	947 (33.1)
Number of samples for season, n (%)	
Autumn (September–November)	766 (26.8)
Spring (March–May)	713 (24.9)
Summer (June–August)	709 (24.8)
Winter (December–February)	675 (23.6)

**Table 2:** Enteric pathogens identified among positive samples tested in the teaching hospital.

Enteric Pathogen Isolation, n (%)	
Bacteria	432 (62.2)
*Clostridioides difficile* toxin A/B-producing	158 (36.6)
Enteropathogenic *Escherichia coli* (EPEC)	121 (28.0)
Enteroaggregative *Escherichia coli* (EAEC)	34 (7.9)
Shiha-like toxin-producing *Escherichia coli* (STEC)	35 (8.1)
*Salmonella enterica* spp.	27 (6.3)
*Campylobacter jejuni*	27 (6.3)
Enterotoxigenic *Escherichia coli* (ETEC)	19 (4.4)
*Yersinia enterocolitica*	11 (2.5)
Viruses	57 (8.2)
Norovirus	30 (52.6)
Sapovirus	15 (26.3)
Adenovirus	5 (8.8)
Astrovirus	4 (7.0)
Rotavirus	3 (5.3)
Fungi	201 (29.0)
*Candida* spp.	197 (98.0)
*Aspergillus* spp.	4 (2.0)
Parasites	4 (0.6)
*Cryptosporidium*	2 (50.0)
*Giardia lamblia*	2 (50.0)
Ward isolation, n (%)	
Outpatients	205 (29.5)
Oncology	182 (26.2)
Gastroenterology	92 (13.3)
Surgery	64 (9.2)
Neurology	37 (5.3)
Cardiovascular Intensive Care Unit	22 (3.2)
Intensive Care Unit	20 (2.9)
General Medicine	15 (2.2)
Hepatology	15 (2.2)
Infectious disease	13 (1.9)
Pediatrics *	11 (1.6)
Cardiology	9 (1.3)
Geriatrics	4 (0.6)
Urology	3 (0.4)
Nephrology	2 (0.3)

* The number of enteric pathogens isolated in the pediatric ward refers only to the 2022–2023 biennium.

## Data Availability

All data are included within the main text.
